# Seasonal Variation in Home Range Sizes and Daily Distance to Ephemeral Surface Water for African Savannah Elephant (*Loxodonta africana*) in Eastern Okavango Panhandle, Northern Botswana

**DOI:** 10.1002/ece3.70758

**Published:** 2025-01-09

**Authors:** Anastacia A. Makati, Anna Songhurst, Emily Bennitt, Gaseitsiwe S. Masunga, Graham McCulloch, Amanda Stronza, Tiroyaone A. Matsika, Frank D. Eckardt

**Affiliations:** ^1^ Okavango Research Institute University of Botswana Maun Botswana; ^2^ Environmental and Geographical Science University of Cape Town Rondebosch South Africa; ^3^ Ecoexist Trust Maun Botswana; ^4^ Department of Zoology University of Oxford Oxford UK; ^5^ Texas A&M University College Station Texas USA; ^6^ Botswana University of Agriculture and Natural Resources Gaborone Botswana

**Keywords:** climate variability, forage, kernel density, land use, NDVI, resource availability, space use

## Abstract

African elephants ( *Loxodonta africana* ) are megaherbivores of the African savannas requiring extensive ranges that can provide critical resources for their survival and reproduction at different spatiotemporal scales. We studied seasonal differences in home range sizes and daily distance to the nearest surface water sources by five male and 10 female African elephants in the eastern Okavango Panhandle in northern Botswana between 2014 and 2017. We hypothesized that (i) elephant home ranges would be larger in the wet than in the dry season (because critical resources tend to be less localized in the wet than in the dry season), (ii) the daily distance of the elephants to the nearest ephemeral surface water sources would be larger in the dry than in the wet season because many of the ephemeral water sources would be dry in the dry season and elephants would start moving towards permanent water sources such as rivers, and lastly (iii) that the differences in elephant home ranges and daily distance to water would differ between sexes. Our results showed that elephant home ranges were larger in the wet than in the dry season, and that they differed between sexes with female elephants having smaller home ranges in the late wet season. The mean daily distance to the nearest ephemeral surface water sources was larger in the dry than in the wet season. There was an inverse relationship between elephants' daily distance to permanent surface water and to ephemeral water sources. The findings indicate the need for large conservation areas and functional connectivity between landscapes to enable the highly mobile savanna elephants to access critical seasonal resources such as water and forage in semi‐arid savannas. Such landscapes are critical, especially in the face of climate change, when high air temperatures and droughts may exacerbate forage and water shortage and intensify human–elephant interactions in surrounding agroecosystems.

## Graphical abstract

We studied seasonal differences in home range sizes and distance to the nearest ephemeral surface water sources by five male and 10 female African elephants in the eastern Okavango Panhandle in northern Botswana between 2014 and 2017. Elephants had larger home ranges in the wet season than in the dry season and the distance to the nearest ephemeral surface water sources was larger in the dry season than in the wet season. The findings indicate the need for large conservation areas and functional connectivity between landscapes to enable the highly mobile savanna elephants to access critical seasonal resources such as water and forage in semi‐arid savannas. Such landscapes are critical, especially in the face of climate change, when high air temperatures and droughts may exacerbate forage and water shortage and intensify human–elephant interactions in surrounding agroecosystems.

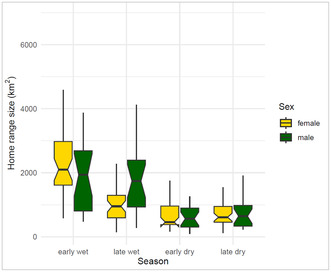

## 1 Introduction

Large African herbivore species such as the African elephant ( *Loxodonta africana* ), blue wildebeest (*Connochaetus taurinus*), and plains zebra ( *Equus quagga* ) are known for their long‐distance seasonal movements undertaken to access essential minerals, forage, and water sources, which often are sparsely distributed over time and space due to seasonally erratic rainfall (Chamaille‐Jammes, Valeix, and Fritz 2007; Duffy et al. 2011). However, the seasonal movements of these herbivores are gradually being restricted or diverted as human developments and activities around and between wildlife areas increase (Naidoo, Angula, et al. 2022). Examples of such anthropogenic activities include expansion of human settlements (Ngene et al. 2017), extensive agriculture, and erection of cordon fences (Naidoo, Beytell, et al. 2022). Because of the dire need to access critical resources between seasons, large herbivore species, and often the most feared by humans such as the African elephant finds itself coming into serious conflict with man, hence increasing calls to reduce its numbers through hunting or removal from agroecosystems (Öhman 2015). In terms of the human–wildlife conflict, the African elephant is the most problematic species causing huge losses in crop production, farm implements and human lives, and consequently high poverty and resentment in the form of poaching by local communities (Songhurst 2017; Schlossberg, Chase, and Griffin 2018). The increasing elephant populations across its ranges and associated human–elephant conflict incidents outside protected areas calls for regular and empirical information on how elephants use their space within and between seasons. The intensification of climate change, which is manifesting in the form of increasing droughts (Van Beest et al. 2011), frequent fires (Nunes et al. 2021), and increasing dispersal especially by elephants (Dejene et al. 2021) will require wildlife managers and land use planners to have regular and adequate information on wildlife movements and distribution to effect pro‐active adaptive wildlife management strategies.

Local movements of an animal species within a range in a particular time period defines its home range, which is simply defined as “an area used by an animal in its daily activities of foraging, mating, and caring for young” (Burt 1943, 351; Powell and Boitani 2012). Because of the need to meet their physiological requirements animals must move from one location to another or between seasonal ranges (Parker, Barboza, and Gillingham 2009). For large herbivores such as the African elephant, which consumes about 150–400 kg of forage and drinks between 50 and 200 L of water a day (Ortega and Eggert 2004), space availability and the ability to move over a wide landscape to utilize supplementary and complementary resources is crucial. However, the availability of large and open landscapes to elephant populations, and hence their homes ranges are increasingly getting smaller across the elephant historical ranges in Asia and Africa. There is therefore a need to monitor these changes in home range sizes and areas covered by elephants as they conduct their daily search for food and mates for social interaction, including reproduction. Continued research will improve the amount and quality of data on elephant home ranges that is currently limited (Shaffer et al. 2019). Despite the huge declines in elephant numbers in some of their historical ranges in some parts of Africa and the provisioning of artificial water points which might have limited their physiological need to move large distances within and between seasons (Chase et al. 2016), there are still some extensive ranges in the world that allow for large home ranges and access to seasonal ranges. Such large landscapes occur in the Kavango Zambezi region in Botswana, Namibia, Zimbabwe, Zambia, and Angola, which has an estimated population of 227,900 individuals (Chase et al. 2016; Thouless et al. 2016; Gross and Heinsohn 2023). Elephants move across this landscape to forage and drink from both ephemeral and permanent surface water sources (Tsalyuk et al. 2019; Pandraud 2022).

African elephants are water dependent and need access to drinking water every 1–3 days (Smit, Grant, and Whyte 2007; Gaugris and Van Rooyen 2010; Dunkin et al. 2013). In environments where water resources are uniformly distributed within the landscape, elephant distribution assumes a similar uniform distribution pattern (Dzinotizei et al. 2018). Where water sources are skewed toward one side of the range, elephants tend to congregate in areas with water (Skarpe et al. 2000). Thus, surface water availability can be a critical factor influencing elephant home range sizes and distance traveled to access resources such as water within and between seasonal ranges (De Beer and Van Aarde 2008). In some elephant conservation areas, especially in semi‐arid countries, artificial provisioning of water to wildlife through pumping out underground water has become a necessary elephant management strategy (Tshipa et al. 2017). It is not only the distribution of waterholes that is critical for elephant space use and distribution but also the quantity and length of time the waterholes continue to provide sufficient water to elephants (Chamaille‐Jammes, Valeix, and Fritz 2007; Branco et al. 2019).

Elephants are heavy consumers of plant matter, eating up to 250 kg of forage daily depending on body weight (Kohi 2013). Although elephants also need nitrogen‐rich plant tissues such as grasses and tree leaves (Mattson Jr 1980), much of their food comprise carbohydrates, mostly fiber and specific‐nutrient sources including leaves, twigs, tree barks, and roots (Ben‐Shahar and Macdonald 2002; Prajapati 2008). Elephant range should, therefore, be able to provide these diverse sources of nutrients to elephants in all seasons as well as at different times of the day. Elephants may need more carbohydrates at certain times of the day to fill up their stomachs and enhance food digestion (Owen‐Smith 1988) and may need protein‐rich or mineral‐rich food at other times of the day or season depending on their physiological requirements (Sach et al. 2019). Female elephants may need more mineral‐rich foods during pregnancy and breastfeeding for bone development of their calves (Takatsu et al. 2017). Home ranges may, therefore, differ between seasons and between sexes of elephants.

In a climate change scenario with reduced precipitation, elephants are expected to gather in locations with increased surface water retention as water sources become limited. With declining precipitation, water availability in numerous natural habitats decreases, prompting elephants to search for the limited remaining areas with accessible water. This concentration on certain areas raises the chances of elephants coming into contact with human settlements that depend on the same water sources for farming, raising animals, and everyday necessities (Kupika et al. 2017). Rising temperatures also contribute to the human–elephant conflict. Elephants rely heavily on water and climate change worsens water scarcity in semi‐arid areas. As water sources decrease, elephants and humans are attracted to areas with water, increasing competition. The joint dependence on scarce water resources, especially in the face of climate change, greatly increases the risk of human–elephant conflicts in these areas. Rainfall is another major underlying factor of elephant distribution as found in studies in Kruger National Park in South Africa (MacFadyen et al. 2019), West Africa, East Africa, and Southern Africa (Dejene et al. 2021). Climate change worsens other impacts described above. The Standardized Precipitation Evapotranspiration Index (SPEI) is a new drought index that helps in detecting, monitoring, and analyzing droughts. This can be used to assess trends in precipitation and temperature. Drought events can be characterized by the SPEI over various temporal scales (Ye et al. 2019).

This study examines the difference in home range sizes between 15 African elephants (5 male and 10 female) in the eastern Okavango Panhandle in northern Botswana across wet and dry seasons between 2014 and 2017. The eastern Okavango Panhandle presents a wide and large landscape which elephant can move freely and where they can access key critical resources needed in different seasons. Botswana has the largest contiguous population of elephants in the world with about 130,000 individuals (Bussière and Potgieter 2023) ranging across the northern part of the country, which falls within the Kavango–Zambezi Transfrontier Conservation Area (KAZA TFCA). The KAZA TFCA is one of the largest conservation areas in Southern Africa transcending the borders of Angola, Botswana, Namibia, Zambia, and Zimbabwe (Songhurst, Mcculloch, and Stronza 2015). Studies indicated that in the eastern Okavango Panhandle, there are about 18,000 elephants occupying over 80% of the area (Pozo et al. 2017; Matsika et al. 2023). The human population is low (16,300 people) (Central Statistics Office, Botswana 2021) and is largely and linearly distributed along the Okavango River which also provides permanent surface water to elephants and people (Pozo et al. 2018).

In this study, we hypothesized that (i) elephant home ranges would be larger in the wet than in the dry season (because forage and water sources tend to be more plentiful and widely distributed in the wet season than in the dry season, and elephant would not be restricted to localized resource areas), (ii) the daily distance of elephant to the nearest ephemeral surface water sources would be larger in the dry than in the wet season as the elephants move further away when ephemeral water sources dry up, and last (iii) the differences in the elephant home ranges and daily distance to water would differ between sexes with female herds showing smaller home range sizes and proximity to surface water. Elephants need forage and water regularly and they must commute between foraging sites and surface water sources to satisfy their nutritional and water requirements. Without any major hindrances, elephants would opt for shorter distances between foraging sites and water sources.

## 2 Materials and Methods

### 2.1 Study Area

**FIGURE 1:**
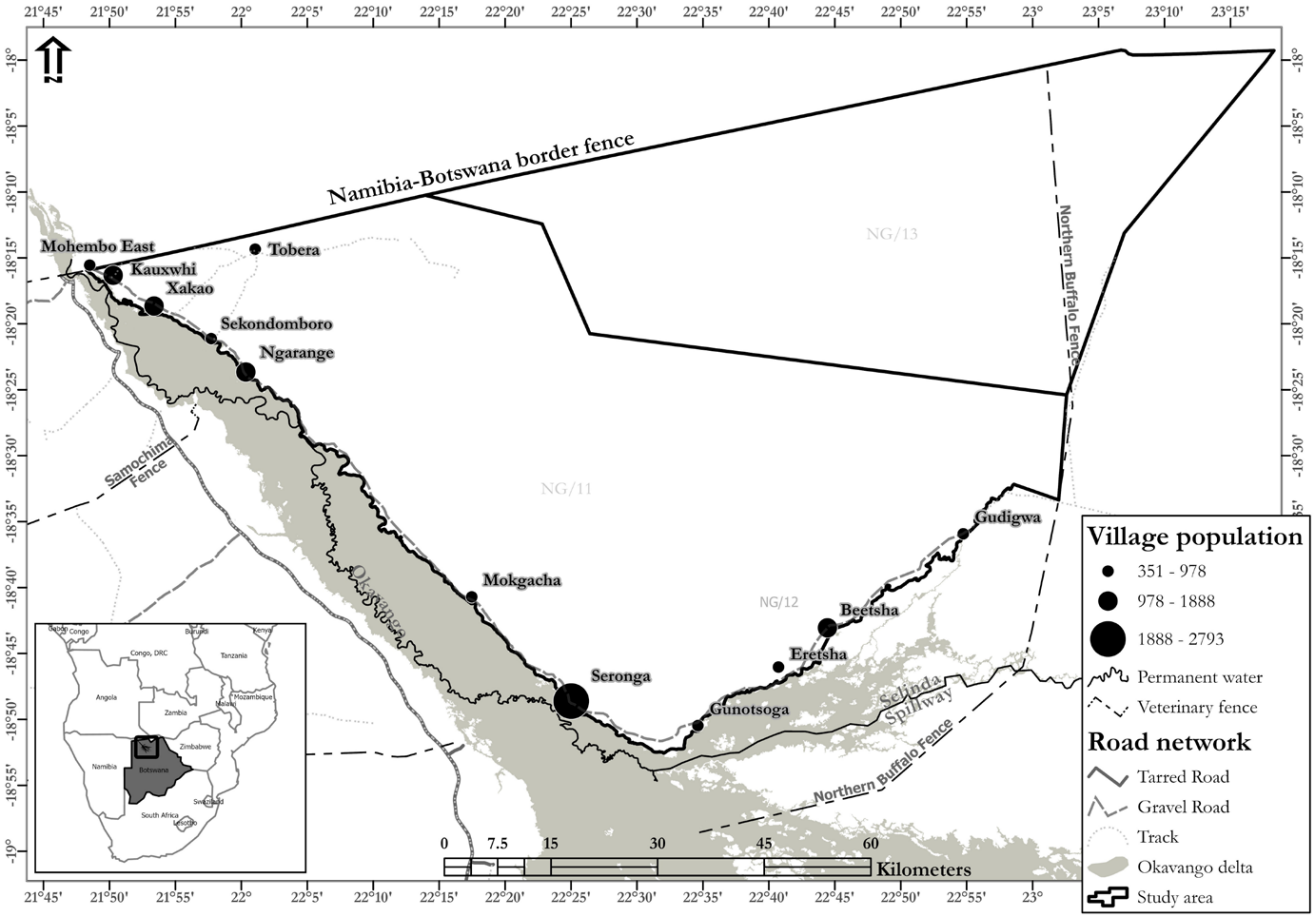
Map of the study area, within Area NG11, where the 12 sampled villages are illustrated according to population size.

The study was conducted in the eastern Okavango Panhandle, northern Botswana from April 2014 to December 2017 (Figure 1). The study area covers an area of 8732 km^2^ and includes three wildlife management areas, NG/11, NG/12, and NG/13. These areas are not fenced and therefore they allow free movement of wildlife across their boundaries. The climate is semi‐arid savanna with strictly seasonal and erratic rainfall falling between November and April. The dry season starts in May–October with the minimum temperatures around 17°C in July, and maximum temperatures reaching 37°C in September and October (Songhurst and Coulson 2014).

The soils in the study area are sandy (arenosols) and support deciduous dry savanna woodlands, open‐tree savannas, shrublands and grasslands on the fossil sand dunes (Hartemink and Huting 2008; Cao 2017). Toward the Okavango River in the west and south, the vegetation is mostly riverine with dense woodlands and floodplain grasslands (Thomas 1991; Ringrose, Vanderpost, and Matheson 2003). The topography is generally flat with a slightly higher elevation of 1068 m in the north, and slopes in the south‐west and south‐east toward the Okavango River which is at 937 m above the sea level. Toward the north and central parts of the eastern Okavango Panhandle are gently undulating and longitudinal east–west fossil sand dunes characteristic of the Kalahari sand region (Gumbricht, Mccarthy, and Merry 2001). The area also has numerous ephemeral pans scattered across the landscape that dry at different times after the rainfall season, although some can hold water for a long duration into the late dry season or into the next rainfall season. Some pans can hold water until August, with some larger pans able to hold until the next rainy season (Chase et al. 2018). Much of the inner and further parts in the north and east have few and no seasonal water pans and are rarely used by water‐dependent animals in the dry season. The Okavango River in the Panhandle of the Okavango Delta is the only source of permanent surface water in the area and is heavily used by water‐dependent wildlife in the dry season, which includes elephants (Songhurst, Mcculloch, and Coulson 2016; Pozo et al. 2018; Chase et al. 2018; Buchholtz et al. 2019).

There are about 18,000 elephants in the eastern Okavango Panhandle as well as large populations of plains zebra ( *Equus quagga* ), sable antelope ( *Hippotragus niger* ), roan antelope ( *Hippotragus equinus* ), greater kudu ( *Tragelaphus strepsiceros* ), and giraffe ( *Giraffa camelopardalis* ). The area also has a good number of wild carnivores such as the African lion ( *Panthera leo* ) and leopard ( *Panthera pardus* ) (Bothma and Walker 2013). There are 14 main villages, supporting 16,300 people, situated along the stretch of the Okavango River (Central Statistics Office, Botswana 2021). Sources of livelihoods are tourism, and pastoral and arable farming (Songhurst, Mcculloch, and Stronza 2015). Human–elephant conflict is intense in NG11 where there is high interaction between agricultural activities and elephants that are accessing water and resources in the Okavango River and Okavango Delta, and consequently raid farmers' crops, damage property, and threaten people's lives (Songhurst 2023).

#### 2.1.1 Elephant Collaring and Tracking

We used Global Positioning System (GPS) collar (Iridium Vectronics) data from 15 elephants (5 males, 10 females) collared in wildlife management areas NG/11, NG/12, and NG13 in the eastern Okavango Panhandle in April 2014. Each collar was set to produce hourly GPS fixes. Individuals were selected using a spotter plane and darted and immobilized from a helicopter. All collaring procedures were supervised by a veterinarian and performed under the research permit EWT 8/36/4 XVII (79), and immobilization permits issued to Ecoexist Trust. To reduce bias toward any specific area within the eastern Okavango Panhandle individual elephants were selected from independent herds. Females were selected based on their body size and age of their calves (> 3 years) if any. Larger individuals were preferred as they were considered mature adults and highly likely to be matriarchs. All collared males were older than 20 years (Songhurst 2014). For this study, we used data from April 2014 to December 2017, and a total of 10,518–30,609 fixes per elephant were downloaded (Table 1).

**TABLE 1:** Data sets used for the study.

Elephant name	ID	Deployment/tracking period	Sex	Age	No. fixes
Pille	14648	22 April 2014–April 2017	Female	20+	30,609
Nare	14649	22 April 2014–April 2017	Female	25	18,361
Mpule	14650	24 April 2014–April 2017	Female	25	27,791
Mbamba	14653	25 April 2014–April 2017	Female	20+	30,260
Rain	14654	24 April 2014–April 2017	Female	20	17,395
Ann	14656	23 April 2014–April 2017	Female	20	14,861
Ebby	14659	24 April 2014–April 2017	Female	16–20	29,977
Koo	14660	23 April 2014–April 2017	Female	20	29,492
Whisper	14661	25 April 2014–April 2017	Female	25	11,866
Amantle	14664	23 April 2014–April 2017	Female	20	10,518
Howard	14646	23 April 2014–April 2017	Male	40+	21,543
Chan	14652	25 April 2014–April 2017	Male	36+	20,734
G	14657	23 April 2014–April 2017	Male	40	19,266
The Bachelor	14658	24 April 2014–April 2017	Male	30	20,502
Whiskey	14662	25 April 2014–April 2017	Male	30	30,144

#### 2.1.2 Normalized Difference Vegetation Index

The Landsat 8 satellite imagery was used to derive Normalized Difference Vegetation Index (NDVI) to determine vegetation greenness which was used in this study as a proxy for forage availability for elephants. NDVI reflects the presence of chlorophyl or green vegetation which herbivores are attracted to meet their nutritional requirements. We used high resolution (30 m) Landsat images that were a composite of images collected over a 16‐day period (United States Geological Survey 2013). The images were processed by the United States Geological Survey (USGS) to produce NDVI images with values. The NDVI values are generated from the Landsat images by determining the ratios of red to near‐infrared reflectance [NDVI = (NIR RED)/(NIR + RED)], where NIR and RED are the spectral reflectance within the near‐infrared and red‐light bands recorded by a satellite's sensor, respectively (Guerschman et al. 2009; Pettorelli et al. 2011). NDVI values range from −1 to +1, with values close to 1 indicating very green or good condition vegetation, values around 0 suggesting less green or sparse vegetation, whereas negative values typically indicate very dry, bad condition vegetation or nonvegetated landscape (Malik, Nasrudin, and Withaningsih 2023). The NDVI values were derived within the location coordinates of the collared elephants to facilitate correlation analysis.

#### 2.1.3 Elevation

The Digital Elevation Model (DEM) data overlapping the locations of the collared elephants were extracted from Space Shuttle Radar Topography Mission (SRTM) platform, which is freely accessible on USGS Earth Explorer platforms (https://earthexplorer.usgs.gov/). Elevation values were extracted from three SRTM tiles of 1‐arc‐second (30 m) resolution that covered the study area. The values were analyzed with other environmental and elephant movement data.

#### 2.1.4 Distances to Water and Settlements

Landsat 8 satellite imagery was used to derive the Automated Water Index (AWEI) from which ephemeral water bodies layers were obtained, whereas locations for permanent water sources and human settlements were obtained from the ODIS database (https://www.ub.bw/services/geographical‐information‐systems‐gis‐laboratory). The aquatic and settlement layers were then overlaid with elephant locations to determine daily distances between them and collared elephants at hourly intervals. Daily distances from the nearest ephemeral and permanent water sources and human settlements were all calculated to the nearest elephant fix.

#### 2.1.5 Droughts

Droughts were determined using Standardized Precipitation Evapotranspiration Index (SPEI) (https://spei.csic.es/) covering a 67‐year period from 1950 to 2017. In this study, we used SPEI 48, which is an indicator of prolonged drought conditions. The SPEI uses qualitative drought severity categories that cover a range from 2 to −2 (Table 2). The range from −2 to 0 reflects the occurrence of drought and the range from 0 to 2 depicts nondrought conditions. The SPEI values less than −1.5 indicate severe to extreme drought conditions.

**TABLE 2:** Drought classification based on SPEI values.

Level	Drought category	SPEI values
0	Nondrought	0 ≤ index
1	Mild drought	−1.0 < index < 0
2	Moderate drought	−1.5 < Index ≤ −1.0
3	Severe drought	−2.0 < Index ≤ −1.5
4	Extreme drought	Index ≤ −2.0

*Source:* Tan, Yang, and Li (2015).

### 2.2 Data Analysis

#### 2.2.1 Home Range Size

The elephant fixes totaling 334,066, were downloaded using the Vectronic GPS PLUS X Collar Manager into Microsoft Excel for processing. This data set was then imported into R statistics version 4.0.1 for visualization and statistical analysis (R Core Team 2013). To ensure data quality, a cleaning process was undertaken. Initially, rows lacking XY coordinates and data falling outside the study period were removed (Songhurst 2014). To eliminate duplicate fixes, the *getDuplicatedTimestamps* function from the “Move” package, in R version 4.0.3 (Kranstauber, Smolla, and Scharf 2020), was employed. GPS collar data were collected over a 4‐year period and were divided into four seasons: early wet (more rains: November to January), late wet (slight rains: February–April), early dry (no rains: May–July), and late dry (high temperatures and no rains: August–October).

The 95% kernel utilization distribution (UD) for each elephant was derived with the kernel density estimation (KDE) method (Van Winkle 1975; Fieberg and Kochanny 2005), from the *adehabitat* package in R version 4.0.3 (Calenge 2006) to allow a comparison of home range sizes between seasons and sex (Benhamou 2011). The reference smoothing parameter (*h_ref*) (Worton 1987; Viana et al. 2018) was used to calculate home ranges and 95% contours (Börger et al. 2006). The smoothing parameter *h_ref* was chosen over least square cross‐validation (LSCV) which is widely used because the latter did not converge with the data set used. Kernel home range estimators were selected over Minimum Convex Polygon (MCP) estimators since the MCP does not reveal the real outline of the home range but considers areas that are infrequently visited identically to those that are often visited. Kernels differentiate intensively used locations more precisely (Worton 1987). From these home ranges, seasonal home range sizes were extracted for every collared individual elephant. To generate complete seasonal home range sizes, we used data from the collars that had consistently recorded GPS fixes throughout the entire season.

The data did not satisfy the assumptions of normality after a Shapiro–Wilk test. The data were then log‐transformed, after which they followed a normal distribution (*W* = 0.99184; *p* value = 0.2043). The effects of season on home range size were analyzed using a linear mixed effects model (LMM) in *R lme4* package (Bates et al. 2015), with the log of home range size as the dependent variable, fixed effects predictors variables were two‐way interaction between season and sex, and individual animal and year as the random effect. The dredge function in the *MuMIn* package was used to run models with all combinations of the predictors and identified the most parsimonious models based on AIC values (Akaike 1974).

#### 2.2.2 Daily Distance to Ephemeral and Permanent Water, NDVI and Settlements

We used ENVI 5.4.1 to preprocess raw Landsat 8 imagery and convert digital numbers into surface reflectance and geometric corrections to reduce distortions and finally produced NDVI images. We then used the *Extract Values to Points* tool in ArcGISPro 3.3 to extract the NDVI values. We further used the same preprocessed Landsat 8 images to delineate ephemeral water sources. Raster pixels were converted into vector files using ArcGIS Pro 3.3, and this allowed us to map and visualize the mapped surface water sources with other shapefiles.

Additionally, we used the *Near Analysis* tool in ArcGIS Pro 3.3 to calculate the daily distances (Environmental Systems Research Institute 2023) to ephemeral water, permanent water, and human settlements and for only three seasons (late wet, early dry, and late dry season).

For the elevation data, the SRTM tiles were mosaiced using *Mosaic to New Raster* tool to merge the images into one mosaic, and the *Extract Values to Points* tool in ArcGIS Pro 3.3 provided DEM values for every elephant fix location.

The individual effects of NDVI, gender, elevation, daily distance to human settlements, and year on the daily distances by elephant were investigated using Generalized Mixed Models (GLMMs) with a gamma distribution in R (*glmmTMB* package) using a log link function for the data (Bolker 2019). Daily distance to ephemeral surface water was used as the dependent variable. Fixed effect predictors included a two‐way interaction between daily distance to permanent water and season, an interaction of time of the day and season, and an interaction of year and season, with an individual animal as a random effect to account for repeated observations and allow for variation among individuals. The year was converted into a factor in R to assess its influence on the daily distance to ephemeral surface water. The dredge function in the *MuMIn* package was also used to run the model with all combinations of the predictors and identified the most parsimonious global models based on AICc values (Akaike 1974; Barton 2009). When more than one model was competitive (ΔAIC < 2), models were averaged using the *model.avg* function from the *MuMIn* package version 4.0.3 (Barton 2009; Zeugner and Feldkircher 2015).

## 3 Results

### 3.1 Home Range Size

**FIGURE 2:**
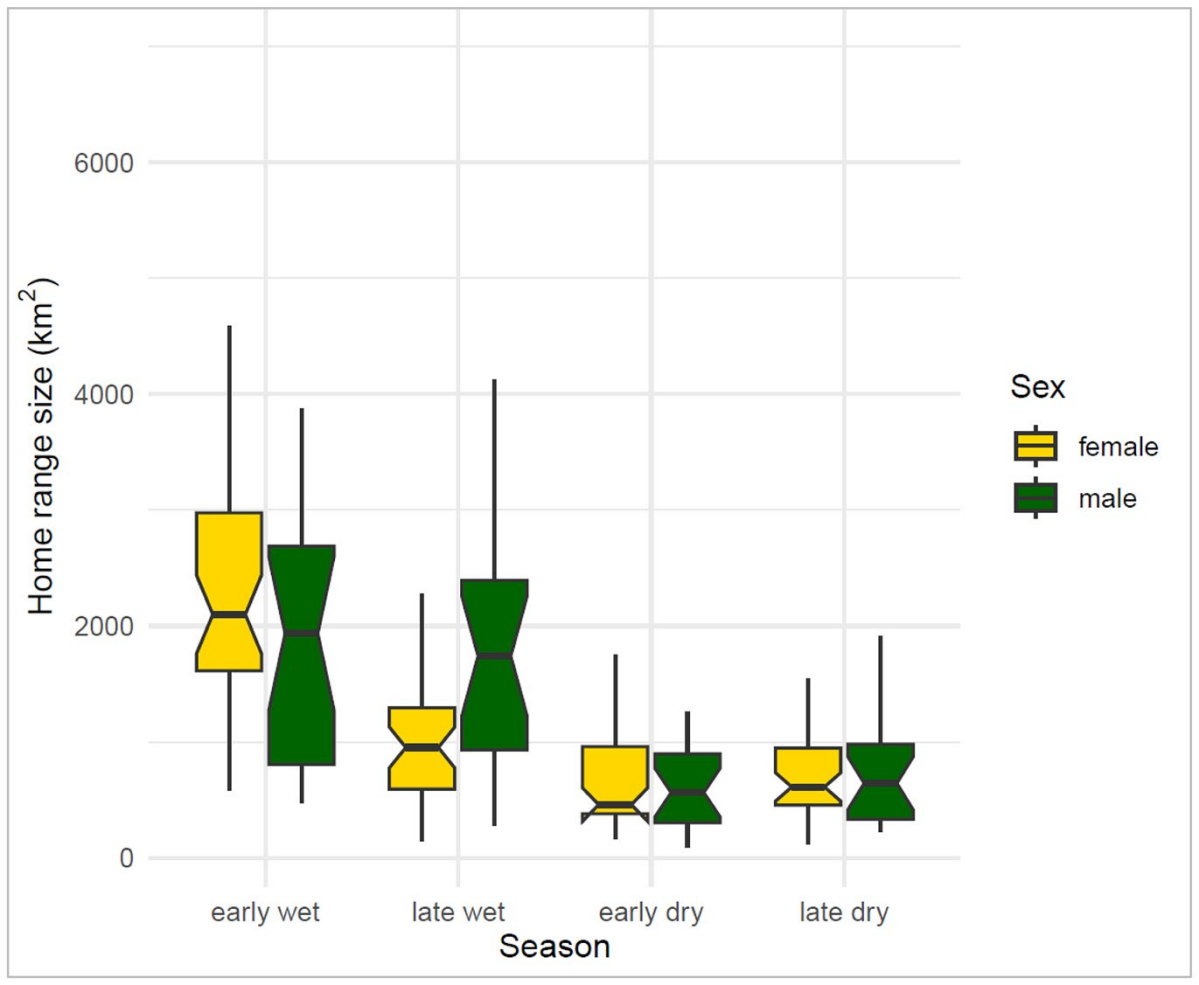
Seasonal home range sizes for male and female elephants in the eastern Okavango Panhandle, Botswana between 2014 and 2017. Home ranges derived from Kernel density estimation using R Statistics version 4.2.1.

The mean seasonal home range size from the collared elephants recorded over the 4‐year period of study was 1256.88 km^2^ (SD 1047.81) and ranged from 500 to 2200 km^2^. The most parsimonious model that explained significant variations in home range sizes included season only (AICc = 464.9, AIC_w_ = 0.720). No other models were competitive (Tables 3 and 4). The mean home range sizes were smaller in the dry season (early and late dry seasons) (500–650 km^2^) than in the wet season (early and late wet seasons) (1100–2200 km^2^) (Figures S1A–D and S2). Female elephants (1000 km^2^) had smaller home range sizes than male elephants (1900 km^2^) in the late wet season (2100 km^2^) (Figure 2).

**TABLE 3:** Home ranges—analysis of variance table with Satterthwaite's method.

	Sum sq.	Mean sq.	NumDF	DenDF	*F* value	Pr(>*F*)	Significance
Season	50.389	16.7963	3	216	54.6981	< 2e‐16	***
Sex	0.000	0.0001	1	13	0.0005	0.9828998	
Season: sex	5.254	1.7514	3	216	5.7036	0.0008934	***

*Note:* Significance levels are denoted as follows: *p* < 0.001: *** (highly significant).for example, < 2e‐16 indicates extremely strong evidence against the null hypothesis (*p* < 0.001), while 0.0008934 is also highly significant at *p* < 0.001.

**TABLE 4:** Candidate models of the effects of season, sex, and their interaction on home range size in African elephants in the eastern Panhandle, northern Botswana, between 2014 and 2017.

Model	(Int)	Season	Sex	Season: sex	df	LogLik	AICc	Delta	Weight
8	7.672	+	+	+	11	−220.877	464.9	0.00	0.720
2	7.565	+			7	−226.317	467.1	2.20	0.239

### 3.2 Daily Distances to Ephemeral Surface Water Source

**FIGURE 3:**
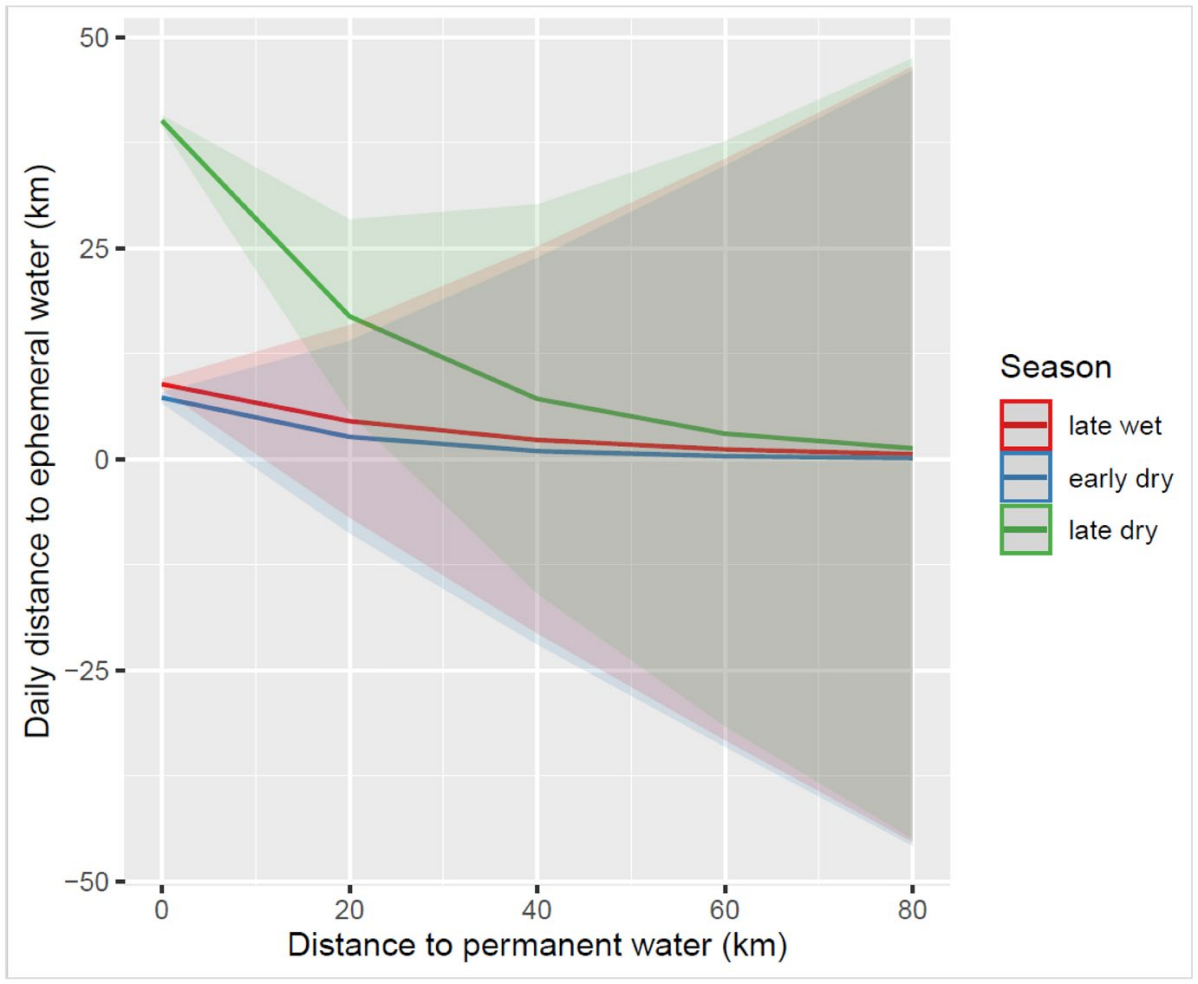
An interaction of permanent water and season to elephant daily distance to ephemeral surface water in the eastern Okavango Panhandle, Botswana from 2014 to 2017. Error bars represent ±1 standard error. Distances derived using *near analysis tool* in ArcGIS Pro 3.0.

Season, permanent water, and year were significant in influencing the elephant's daily distances to ephemeral surface water sources (AICc = 1,782,855, AIC_w_ = 0.329; Tables 5, 6, 7). The most parsimonious model included interaction between season and permanent water and between season and year (AICc = 1,782,855, AIC_w_ = 0.282) (see Tables 5 and 7). No other models were competitive. Regarding seasonal differences, elephants were closer both to the ephemeral water and permanent sources in the late wet and early dry season than in the late dry season (Figure 3). However, in the late dry season, elephants were further from ephemeral water sources by over 30 km.

**TABLE 5:** Table coefficient.

Coefficient	Estimates	Conf. int (95%)	*p*
(Intercept)	0.00	0.00–0.00	**< 0.001**
NDVI	0.28	0.27–0.29	**< 0.001**
Gender (male)	1.13	0.97–1.32	0.114
Elevation	1.03	1.03–1.03	**< 0.001**
Distance to settlements	1.03	1.03–1.03	**< 0.001**
Year (2015)	3.42	3.29–3.55	**< 0.001**
Year (2016)	3.47	3.34–3.60	**< 0.001**
Year (2017)	0.65	0.62–0.67	**< 0.001**
Season (early dry)	0.82	0.78–0.85	**< 0.001**
Season (late dry)	4.53	4.34–4.72	**< 0.001**
Distance to permanent water	0.97	0.97–0.97	**< 0.001**
Time of day (night)	1.00	0.98–1.01	0.688
Season (early dry): distance to permanent water	0.98	0.98–0.98	**< 0.001**
Season (late dry): distance to permanent water	0.99	0.99–0.99	**< 0.001**
Season (early dry): time of day (night)	1.01	0.99–1.03	0.343
Season (late dry): time_of_day (night)	1.00	0.98–1.01	0.658
Year (2015): season (early dry)	1.37	1.32–1.43	**< 0.001**
Year (2016): season (early dry)	2.81	2.69–2.92	**< 0.001**
Year (2017): season (early dry)	4.53	4.34–4.71	**< 0.001**
Year (2015): season (late dry)	0.49	0.47–0.51	**< 0.001**
Year (2016): season (late dry)	0.33	0.32–0.34	**< 0.001**
Year (2017): season (late dry)	0.73	0.70–0.76	**< 0.001**
*Random effects*			
*σ* ^2^	0.76		
*τ*_00 animal ID_	0.02		
ICC	0.03		
*N*_animal ID_	15		
Observations	246,598		
Marginal *R* ^2^/conditional *R* ^2^	0.543/0.555		

*Note:* Significance levels for bold values *p* < 0.001: Shows a highly significant effect of the predictor, strongly rejecting the null hypothesis.

**TABLE 6:** Candidate models of the effects of NDVI, gender, elevation, distance to settlements, year, and an interaction of season and distance to permanent water; an interaction of season and time of the day and an interaction of season and year on daily distance traveled to ephemeral surface water in African elephants in the eastern Panhandle, northern Botswana, between 2014 and 2017.

Model	(Int)	Distance to permanent water * season	Season * time of the day	Season * year	Distance to permanent water	Distance to settlements	Elevation	Gender	NDVI	Season	Time_of_day	Year	df	LogLik	AICc	Delta	AIC_w_
1472	−22.97	+		+	−0.03409	0.02766	0.02593	+	−1.271	+		+	21	−891406.5	1,782,855	0.00	0.329
1464	−22.92	+		+	−0.03409	0.02766	0.02593		−1.270	+		+	20	−891407.6	1,782,855	0.31	0.282
1536	−22.97	+		+	−0.03409	0.02766	0.02593	+	−1.271	+	+	+	22	−891406.4	1,782,857	1.86	0.130
1528	−22.92	+		+	−0.03409	0.02766	0.02593		−1.271	+	+	+	21	−891407.5	1,782,857	2.17	0.111
2048	−22.96	+		+	−0.03409	0.02765	0.02593	+	−1.272	+	+	+	24	−891404.9	1,782,858	2.83	0.080
2040	−22.91	+		+	−0.03409	0.02765	0.02592		−1.271	+	+	+	23	−891406.0	1,782,858	3.14	0.068

**TABLE 7:** Table showing averaged model selection for elephants traveling to the nearest ephemeral water source.

	2.5%	97.5%
Cond (Int)	−23.525919197	−22.374740305
Cond (distance to permanent water)	−0.034695709	−0.033484375
Cond (distance to settlements)	0.026995799	0.028322144
Cond (elevation)	0.025353350	0.026511396
Cond (gender male)	−0.029814585	0.278181863
Cond (NDVI)	−1.318866118	−1.222381302
Cond (season early dry)	−0.240349086	−0.159373388
Cond (season late dry)	1.467433609	1.548798641
Cond (year 2015)	1.190707143	1.266328176
Cond (year 2016)	1.204858479	1.281492732
Cond (year 2017)	−0.472615793	−0.396395755
Cond (distance to permanent water * season early dry)	−0.017556489	−0.016178595
Cond (distance to permanent water * season late dry)	−0.009910269	−0.008227672
Cond (season early dry * year 2015)	0.277767878	0.359029665
Cond (season late dry * year 2015)	−0.752478512	−0.671533357
Cond (season early dry * year 2016)	0.991042899	1.073208917
Cond (season late dry * year 2016)	−1.152175249	−1.070242915
Cond (season early dry * year 2017)	1.468892373	1.550571704
Cond (season late dry * year 2017)	−0.357596744	−0.276078845
Cond (time of day night)	−0.007736439	0.005238525

**FIGURE 4:**
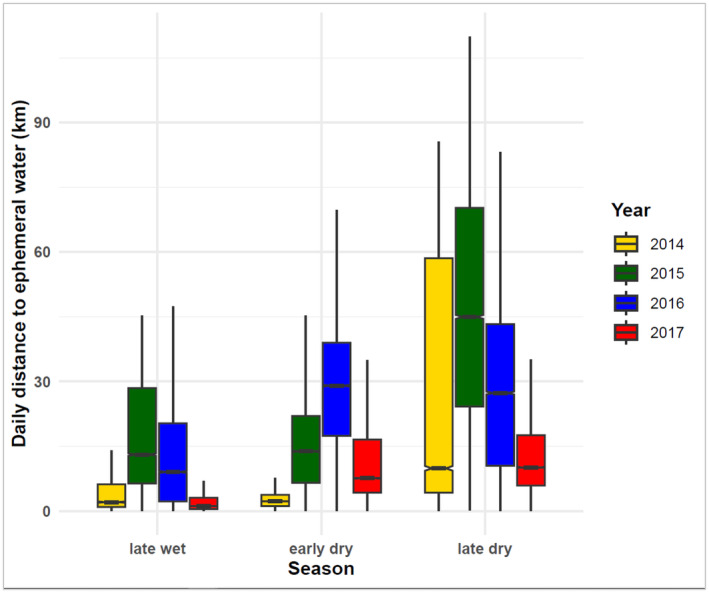
An interaction of season and year to elephant daily distance to ephemeral surface water in the eastern Okavango Panhandle, Botswana from 2014 to 2017. Error bars represent ±1 standard error. Distances derived using ‘near analysis tool’ in ArcGIS Pro 3.0.

When the elephant's daily distance to permanent water increases, the daily distance to ephemeral surface water decreases and vice versa. The daily distance to ephemeral water is larger (35 km) in the late dry season when elephants are at permanent water sources than in the late wet and early dry seasons. In terms of yearly differences, elephant daily distances from the ephemeral surface water were larger in 2015 and 2016 than in other years and were pronounced in the dry season (early and late dry seasons) (Figure 4) (Figures S6 and S7).

### 3.3 Normalized Difference Vegetation Index

There was an inverse relationship between elephant daily distance to ephemeral water and NDVI, indicating that the NDVI values were higher at a closer proximity to the ephemeral water sources than further away (Figure S3).

### 3.4 Elevation

The increase in elevation within the study area showed an exponential increase in the elephant daily distance from ephemeral water sources, indicating that high elevations increase the elephant daily distance to ephemeral water sources (Figure S4). At lower altitudes (960 m), the daily distances to ephemeral water ranged between 5 and 10 km but exponentially increased with increasing elevation.

### 3.5 Distance to Human Settlements and Permanent Water

A near exponential relationship was observed between the daily distance to nearest permanent water source and the daily distance to human settlements. As the daily distance to human settlements increases, the daily distance to ephemeral surface water increases exponentially (Figure S5). On the contrary, as the daily distance to permanent water increased, there was an exponential decrease in the daily distance to the ephemeral surface water sources (Figure S7).

### 3.6 Droughts

The assessment of SPEI48 for the study area indicated that there was a drought from January 2015 to December 2017 (Figure S9A,B). SPEI48 values for 2014 were larger than zero indicating nondrought conditions, whereas for the period between January 2015 and 2016, SPEI48 was below zero, indicating drought conditions. The SPEI48 was less than −1.5 from February 2016 to February 2017, indicating severe to extreme drought conditions. The drought conditions became less severe from March 2017 to the end of the study period.

## 4 Discussion

### 4.1 Home Range Sizes

This study hypothesized that (i) elephant home ranges would be larger in the wet season than in the dry season on the basis that critical resources are abundant and widely distributed over a larger area in the wet season than in the dry season and that elephants would not be restricted to small localities that offer the much‐needed resources. Our study found that elephants had larger home range sizes in the wet season than in the dry season. Generally, resource availability is critical in determining the distribution and abundance of megaherbivore populations in the African savannas (McNaughton 1985; Fryxell 1991; Fynn and Bonyongo 2011). Surface water and forage availability are key determinants of elephant movement and home range size (De Beer and Van Aarde 2008; Loarie, Van Aarde, and Pimm 2009; Cushman, Chase, and Griffin 2005; Cushman 2010). African elephants are water‐dependent and must drink regularly, usually every 1–3 days to maintain their body water requirements and facilitate thermoregulation (Hidden 2009; Mills et al. 2018; Wato et al. 2018). Elephants are also heavy carbohydrate consumers and can ingest over 250 kg of forage daily (Ben‐Shahar and Macdonald 2002; Prajapati 2008; Kohi 2013). Where these quantities of food and surface water sources are widely and abundantly available, elephants can move around widely and cover long daily distances without finding the need to go back to the original location (Adams, Leggett, and Chase 2021), and this may explain why in this study the home ranges were larger in the wet season than in the dry season. Our findings are similar to those found in Chobe in north‐eastern Botswana (Chase 2007; Adams 2016), and other semi‐arid countries where elephant home ranges were consistently larger in the wet season than in the dry season (Western and Lindsay 1984; Osborn and Parker 2003; Galanti et al. 2006; Kikoti 2009; Ngene et al. 2017; Mlambo et al. 2021). However, the home range sizes in our study were much larger than those observed in Kwando (149.68–685.81 km^2^) and Chobe National Park (800–4000 km^2^) in northeastern Botswana. The reason for these large differences in home range sizes of elephants between regions is unknown, but it could be due to challenges relating to access to permanent water sources and forage‐rich habitats which can significantly limit elephant home range sizes. Poaching and hunting can also create a landscape of fear which can confine elephant population to certain parts of their range (Maisels et al. 2013; Schlossberg, Chase, and Griffin 2018). The eastern Okavango Panhandle area is vast with limited human presence, and numerous ephemeral pans that can sufficiently supply ample water to elephants during the wet season. The vastness of the study area limited human presence and relatively adequate forage in the area in the wet season can allow elephants to move widely and freely without fears of human disturbance in the form of poaching. Although habitat type and terrain can also affect elephant movements (Nellemann, Moe, and Rutina 2002) and home range sizes (Thouless 1995; Schulte 2000; Nellemann, Moe, and Rutina 2002; Bohrer et al. 2014; Adams, Leggett, and Chase 2021), such information is limited in the study area. The eastern and northern boundaries of our study area are marked by a veterinary cordon fence that has been found to impede elephant movements between Namibia and Botswana (Naidoo, Beytell, et al. 2022). In this study, we observed cross‐border movements of some collared male elephants from the study area into Namibia and Angola and back to Botswana. This cross‐border movement showed some permeability of the veterinary fence to male elephants and a chance for the elephants to move further and exploit critical resources available within the larger Kavango Zambezi Transfrontier Conservation Area (KAZA TFCA) (Naidoo et al. 2024).

### 4.2 Distance to Ephemeral and Permanent Water, NDVI, Elevation, and Human Settlements

We hypothesized that the daily distance of elephants to the nearest ephemeral surface water sources would be larger in the dry (early and late dry seasons) than in the wet season (early and late dry seasons). Our findings indicated a significant difference in the daily distance of the collared elephants to the nearest ephemeral surface water between seasons, years, and permanent water. Daily distance to the nearest ephemeral surface water was larger in the late dry season than in the late wet season, and this supported the hypothesis that we proposed. The inverse relationship between the daily distance to ephemeral water and the daily distance to permanent water indicates that elephants moved to the permanent water sources in the late dry season when many of the ephemeral points in the study area had dried up. The results indicate that elephants moved long distances from ephemeral water points in the early dry season to converge and become sedentary within a short distance (0–35 km) of permanent water sources in the dry season. Elephants need to travel long distances to be able to reach the critical resources they need to survive and reproduce (Bucciarelli et al. 2024), and this will normally happen at the start of the dry season. These movements are common in semi‐arid environments where forage resources and water availability are generally scarce in quantity and space. However, in the wet season when resources are abundant and widely distributed, elephant movements can be extensive as well because elephants are naturally nomadic (Diaz et al. 2021; Bucciarelli et al. 2024). Male elephants tend to move long distances in the wet season as they are not usually walking young calves and can cover long distances and explore new areas without being hindered by young calves, water availability, or threatened by predation (Ngene et al. 2010; Lee 2011; Mills et al. 2018; Wato et al. 2018). Our study demonstrated two phases of long‐distance movements performed by elephants in our study area. In our study area, elephants moved long distances at the start of the dry season, usually between April and June (Songhurst 2012; Songhurst, Mcculloch, and Stronza 2015; Pozo et al. 2018), away from the ephemeral water sources when these sources dried up, and in doing so, they moved closer to the permanent water sources (Buchholtz et al. 2021). In Chobe National Park, studies found that elephants congest around the Chobe River provides permanent water sources in the dry season and move up to 10 km further from the river daily to browse in the woodlands (Skarpe et al. 2004). Elephants can travel over 70 km every 2–4 days from a wet season range to a dry season range to drink from a permanent water source, and those that are frequent drinkers can reside within 10–40 km from the water source (Viljoen 1989; Skarpe et al. 2004; Wato et al. 2018). Similar observations were found in the forest elephants in the tropical forests of Gabon (Mills et al. 2018). Our study found that in the dry season, elephants covered a large daily distance of 35 km to meet their daily requirements. The daily distance to the nearest permanent water source would be small but very large to the ephemeral water sources. When seasons change from dry to wet, elephants embark again on a long‐distance movement away from the permanent water source to the ephemeral water sources. The movement and presence of elephants around ephemeral water sources were not detrimental to forage quality or greenness as measured by NDVI. Greener or vegetated areas occurred closer to the ephemeral water sources than away, an observation that was not expected since wildlife congestion around water holes has been found to create biospheres or degraded areas around the water holes. However, since NDVI was recorded during the wet season when herbivory is not yet localized, the more greenery vegetation around the waterholes could be a result of a low water table at lower elevations (Tashiya 2023) and increased soil fertilization by dung and urine deposited by elephants and other wildlife species when they come to drink (Clegg 1999; Clegg and O'Connor 2017).

### 4.3 Droughts and Elevation

The analysis of the years in the study period indicates that elephants stayed closer to ephemeral waterholes in 2014 and 2017, which were better years in terms of drought conditions than in 2015 and 2016, which experienced severe droughts. Our other results also showed that in dry seasons, elephants stayed away from the ephemeral water sources but closer to permanent water sources. The daily distance from the ephemeral water sources was larger in years with severe drought conditions than in years with normal dry season conditions. Elephants likely moved to the Okavango River in the eastern and southern boundary and the Kwando River in the far eastern side of the study area, which provide permanent surface water to wildlife in the study area (Buchholtz et al. 2021).

Often large‐bodied or migratory wildlife would prefer areas at lower elevations than areas at higher elevations to save on the energy they have (Nellemann, Moe, and Rutina 2002). Other additional benefits of traveling along low‐elevation terrain are that these areas often have low water table and can support numerous waterpools and dense green vegetation that offers nutrient‐rich forage to wildlife (Berti et al. 2023). Our results indicate that elephants stayed away from highly elevated areas as shown by their large daily distances from ephemeral water sources as the elevation increased. Thus, elevation is a significant landscape factor influencing the distribution and movement patterns of elephants in relation to water availability.

## 5 Conclusion

Elephant home range sizes were larger in the wet season than in the dry season and the daily distance of elephants to the nearest ephemeral water points was larger in the dry season than in the wet season. Sex was influential in determining the home range sizes with female elephants having smaller home ranges than male counterparts in the wet season. Other environmental factors that influenced daily distances from ephemeral water sources were elevation, NDVI and daily distance to human settlements. Informed and integrated land use planning that considers seasonal variations in elephant home range sizes and access to critical seasonal ranges at a landscape scale should be a priority when developments are planned in elephant conservation areas. We recommend that priorities be given to conservation initiatives such as transboundary conservation areas, such as the KAZA TFCA, that provide landscape connectivity and adequate conservation area for large and seasonal mobile wildlife to access critical resources between seasons. Such large and open landscapes are critical, in the face of climate change, when high temperatures and droughts may exacerbate forage and water shortage and force wildlife to move out of their normal habitats and consequently increasing human–elephant conflict in surrounding human‐dominated landscapes.

## Author Contributions

**Anastacia A. Makati:** conceptualization (lead), data curation (lead), formal analysis (lead), funding acquisition (equal), investigation (lead), methodology (lead), project administration (lead), resources (lead), software (lead), supervision (lead), validation (lead), visualization (lead), writing – original draft (lead), writing – review and editing (lead). **Emily Bennitt:** conceptualization (equal), data curation (supporting), formal analysis (equal), funding acquisition (supporting), investigation (supporting), methodology (supporting), project administration (supporting), resources (supporting), software (supporting), supervision (supporting), validation (supporting), visualization (supporting), writing – original draft (supporting), writing – review and editing (supporting). **Anna Songhurst:** conceptualization (supporting), data curation (supporting), formal analysis (supporting), funding acquisition (lead), investigation (supporting), methodology (supporting), project administration (supporting), resources (equal), software (supporting), supervision (equal), validation (equal), visualization (supporting), writing – original draft (supporting), writing – review and editing (equal). **Gaseitsiwe S. Masunga:** conceptualization (supporting), data curation (supporting), formal analysis (supporting), funding acquisition (supporting), investigation (supporting), methodology (supporting), project administration (supporting), resources (supporting), software (supporting), supervision (supporting), validation (equal), visualization (supporting), writing – original draft (equal), writing – review and editing (equal). **Graham McCulloch:** conceptualization (supporting), data curation (supporting), formal analysis (supporting), funding acquisition (lead), investigation (supporting), methodology (supporting), project administration (supporting), resources (supporting), software (supporting), supervision (supporting), validation (supporting), visualization (supporting), writing – original draft (supporting), writing – review and editing (supporting). **Amanda Stronza:** conceptualization (supporting), data curation (supporting), formal analysis (supporting), funding acquisition (supporting), investigation (supporting), methodology (supporting), project administration (supporting), resources (supporting), software (supporting), supervision (supporting), validation (supporting), visualization (supporting), writing – original draft (supporting), writing – review and editing (supporting). **Tiroyaone A. Matsika:** conceptualization (supporting), data curation (supporting), formal analysis (supporting), funding acquisition (supporting), investigation (supporting), methodology (supporting), project administration (supporting), resources (supporting), software (supporting), supervision (supporting), validation (supporting), visualization (supporting), writing – original draft (supporting), writing – review and editing (supporting). **Frank D. Eckardt:** conceptualization (equal), data curation (supporting), formal analysis (supporting), funding acquisition (supporting), investigation (supporting), methodology (supporting), project administration (supporting), resources (supporting), software (supporting), supervision (supporting), validation (supporting), visualization (supporting), writing – original draft (supporting), writing – review and editing (supporting).

## Conflicts of Interest

The authors declare no conflicts of interest.

## Supporting information


**Figure S1.** (A–D) Seasonal home ranges for African elephant (year 2014–2017) in the eastern Okavango Panhandle, northern Botswana.
**Figure S2.** The effect of season on home range size in the eastern Okavango Panhandle.
**Figure S3.** The effect of NDVI on distance to the nearest ephemeral surface water.
**Figure S4.** The effect of elevation on daily distance to the nearest ephemeral surface water.
**Figure S5.** The effect of settlements on daily distance to ephemeral surface water.
**Figure S6.** The effect of year on daily distance to ephemeral surface water.
**Figure S7.** The effect of season on daily distance to ephemeral surface water.
**Figure S8.** The effect of permanent water on daily distance to ephemeral surface water.
**Figure S9.** (A, B) SPEI 48—representing hydrological droughts. Global Drought Monitor online portal; https://spei.csic.es/map/maps.html and is available at a one‐degree spatial resolution for the period 2014–2017 covering time series at cell −18.25° south and 23.75° east.

## Data Availability

Data are stored at Ecoexist Trust offices in Maun and deposited at MOVEBANK; https://datarepository.movebank.org/home.
