# Supplementary material for: Seasonal Variation in Home Range Sizes and Daily Distance to Ephemeral Surface Water for African Savannah Elephant (Loxodonta africana) in Eastern Okavango Panhandle, Northern Botswana
Source: Ecol Evol. 2025 Jan 9;15(1):e70758. doi: 10.1002/ece3.70758 (PMC11712680; doi:10.1002/ece3.70758)
Supplement: Supplementary file 1 — Figure S1. (A–D) Seasonal home ranges for African elephant (year 2014–2017) in the eastern Okavango Panhandle, northern Botswana. Figure S2. The effect of season on home range size in the eastern Okavango Panhandle. Figure S3. The effect of NDVI on distance to the nearest ephemeral surface water. Figure S4. The effect of elevation on daily distance to the nearest ephemeral surface water. Figure S5. The effect of settlements on daily distance to ephemeral surface water. Figure S6. The effect of year on daily distance to ephemeral surface water. Figure S7. The effect of season on daily distance to ephemeral surface water. Figure S8. The effect of permanent water on daily distance to ephemeral surface water. Figure S9. (A, B) SPEI 48—representing hydrological droughts. Global Drought Monitor online portal; https://spei.csic.es/map/maps.html and is available at a one‐degree spatial resolution for the period 2014–2017 covering time series at cell −18.25° south and 23.75° east. [file ECE3-15-e70758-s001.docx]

**Supporting Information**

Seasonal home ranges for African elephant (Year 2014-2017) in the eastern Okavango Panhandle, northern Botswana


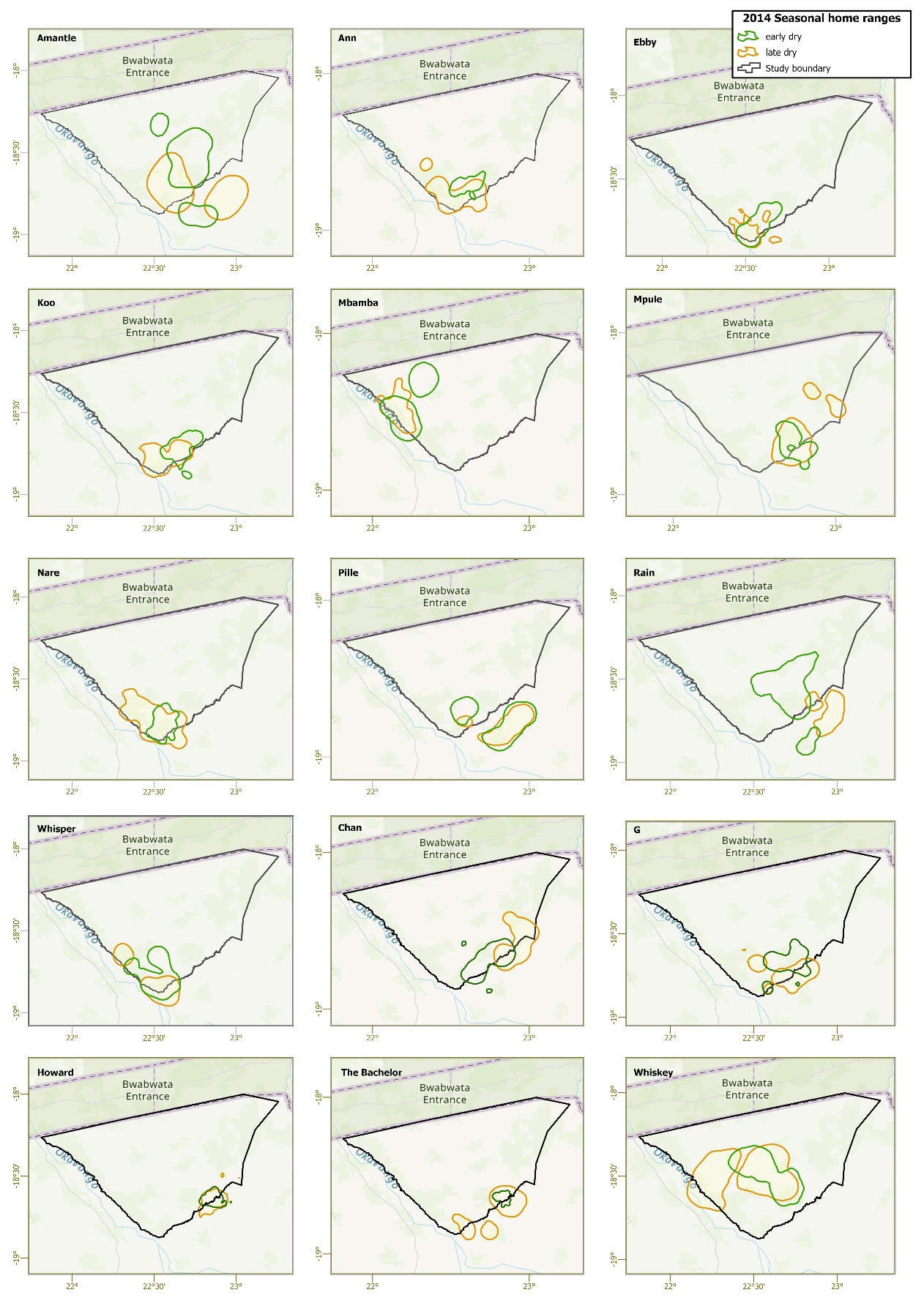


**A**


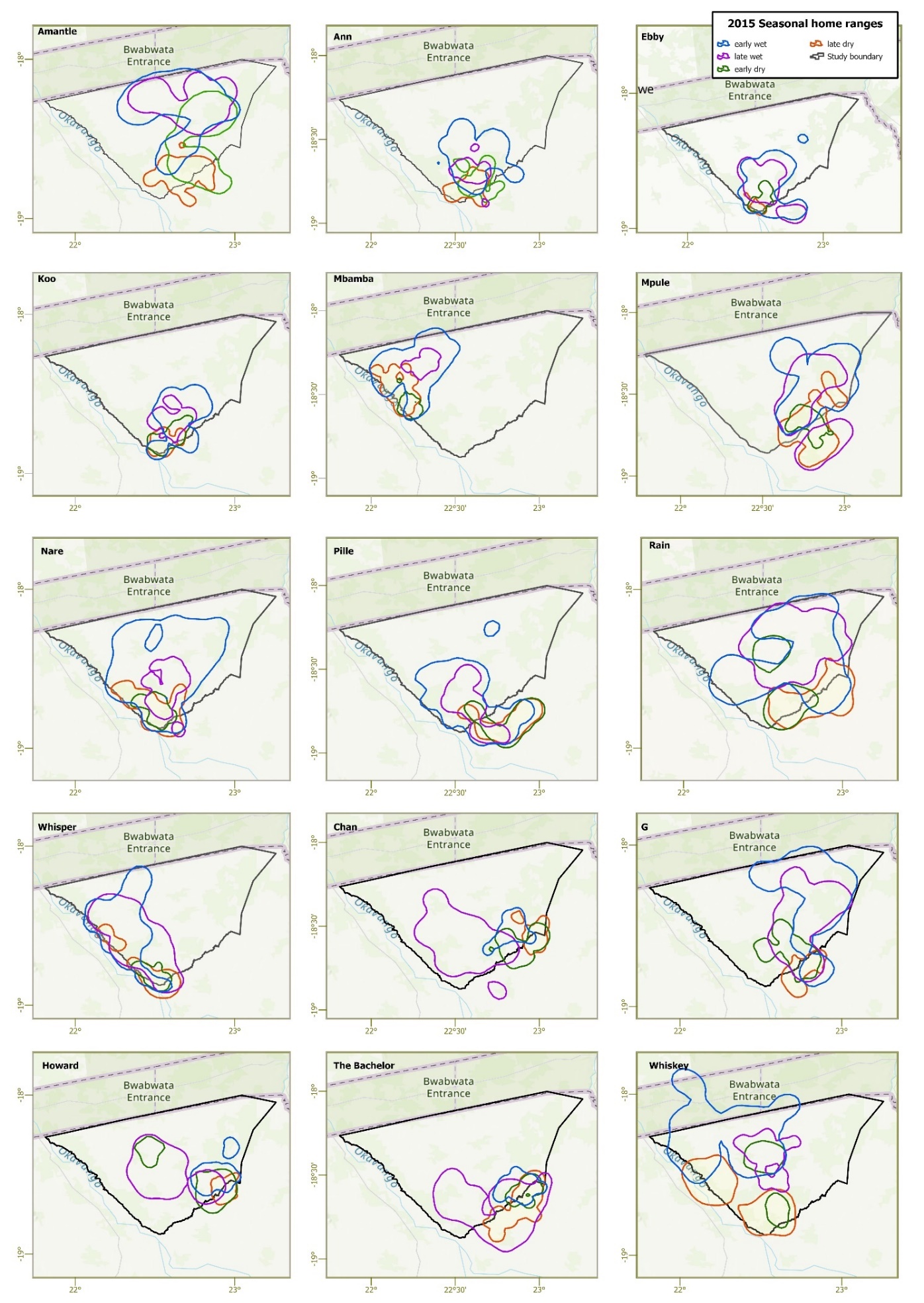


**B**


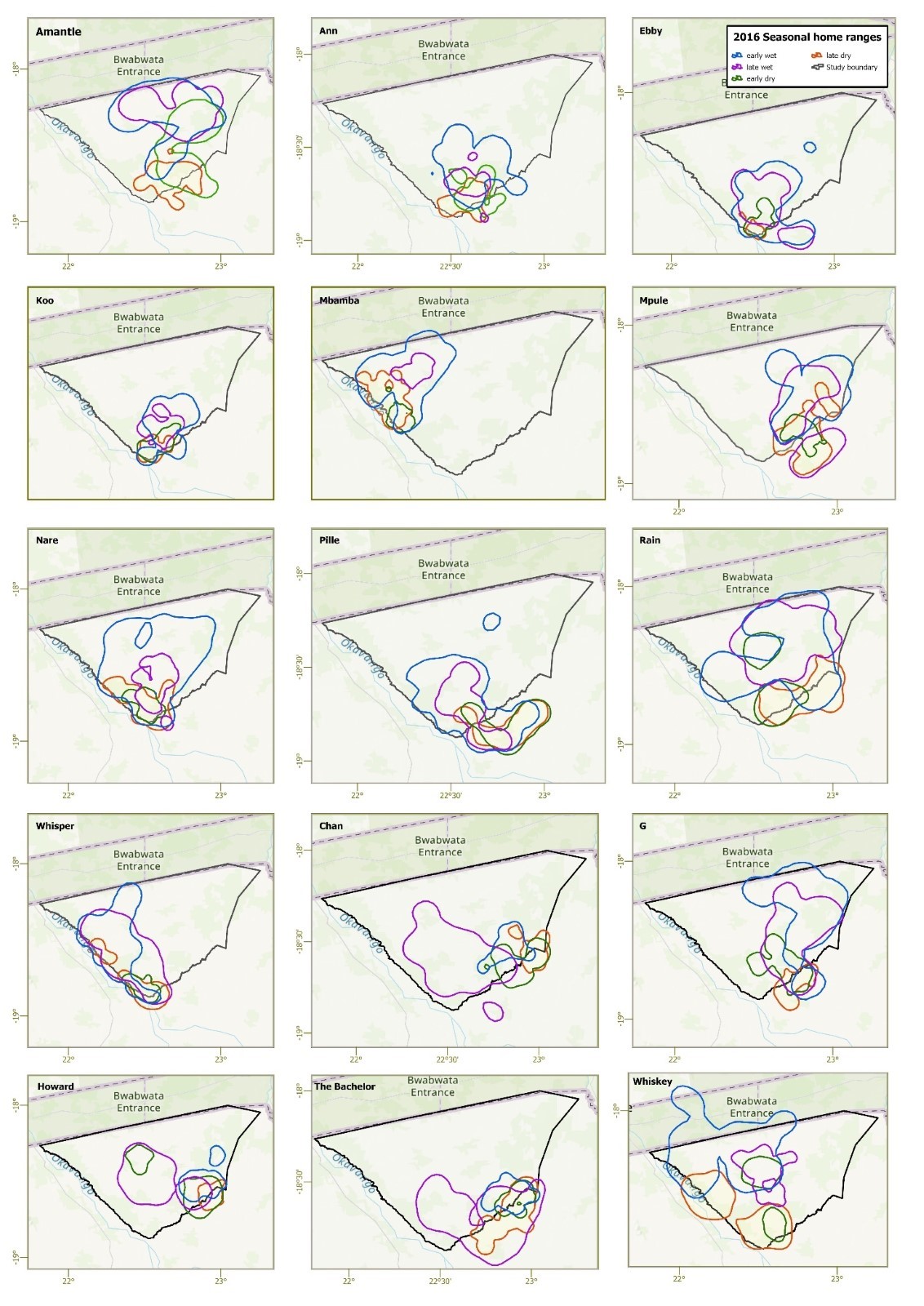


C


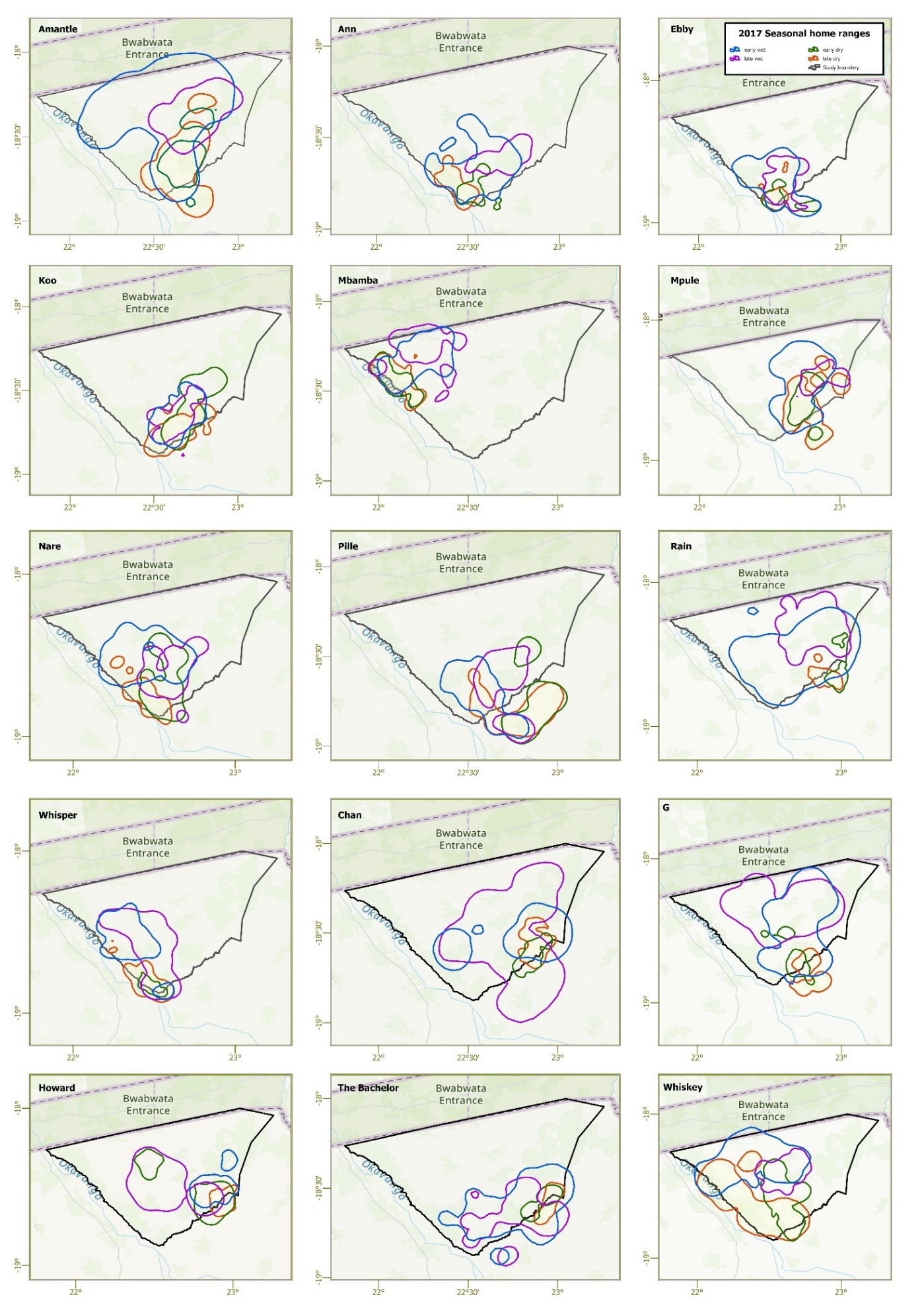


**Figure S1. A-D**

**D**


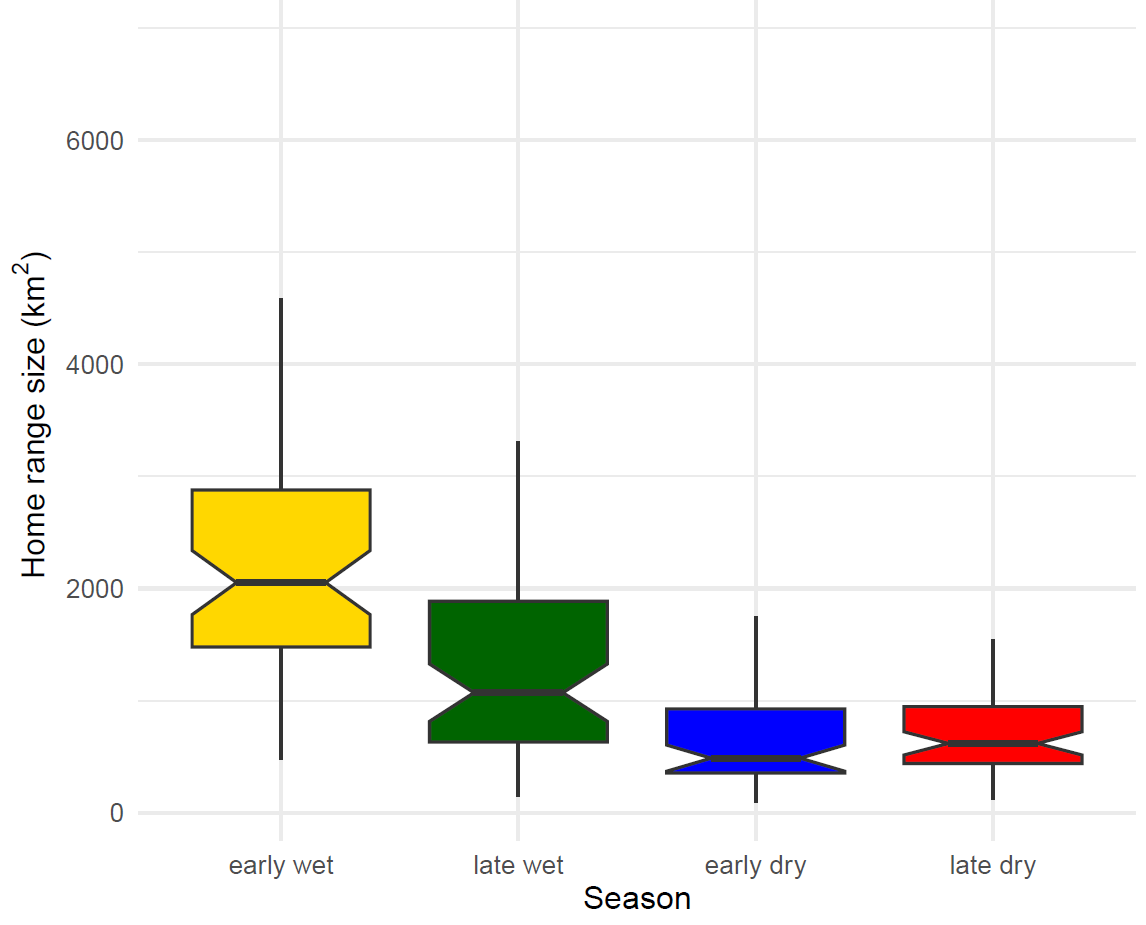


**Figure S2**


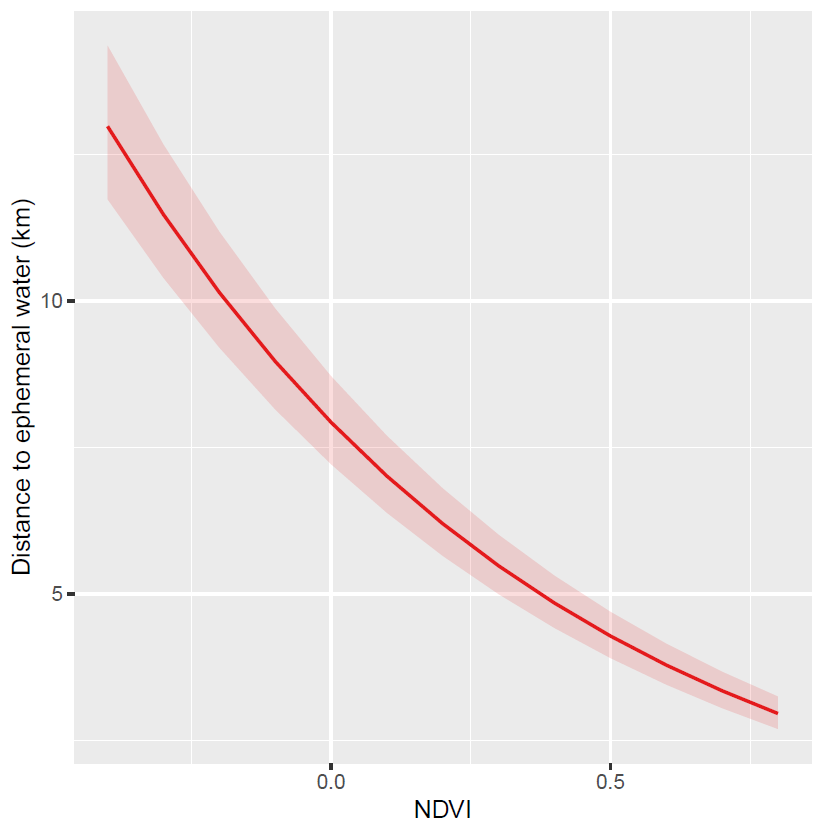


**Figure S3**


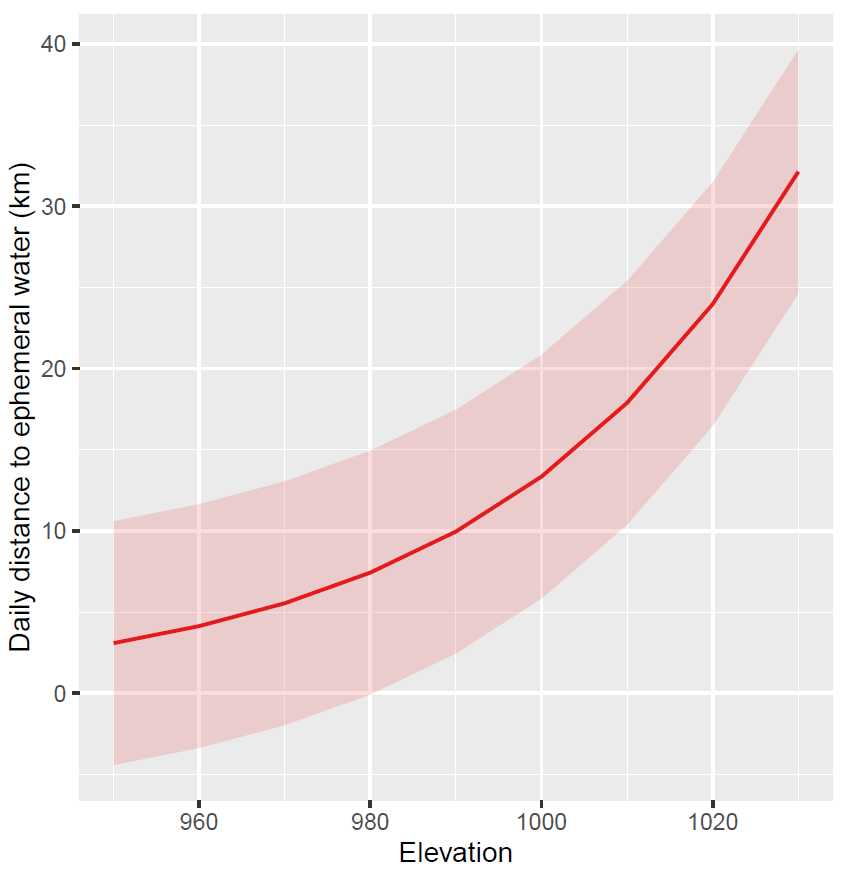


**Figure S4**


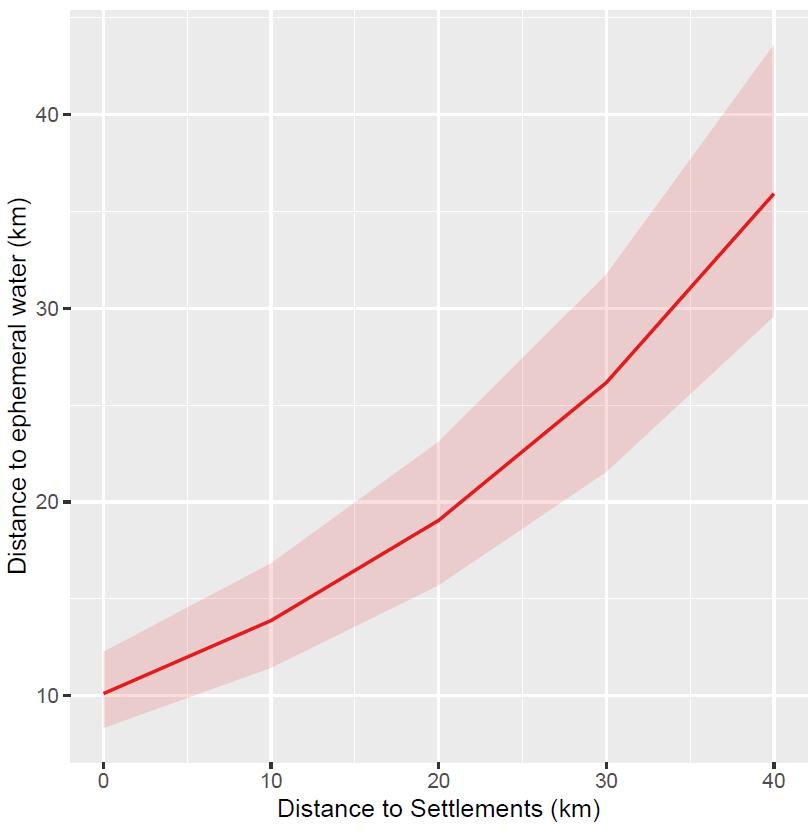


**Figure S5**


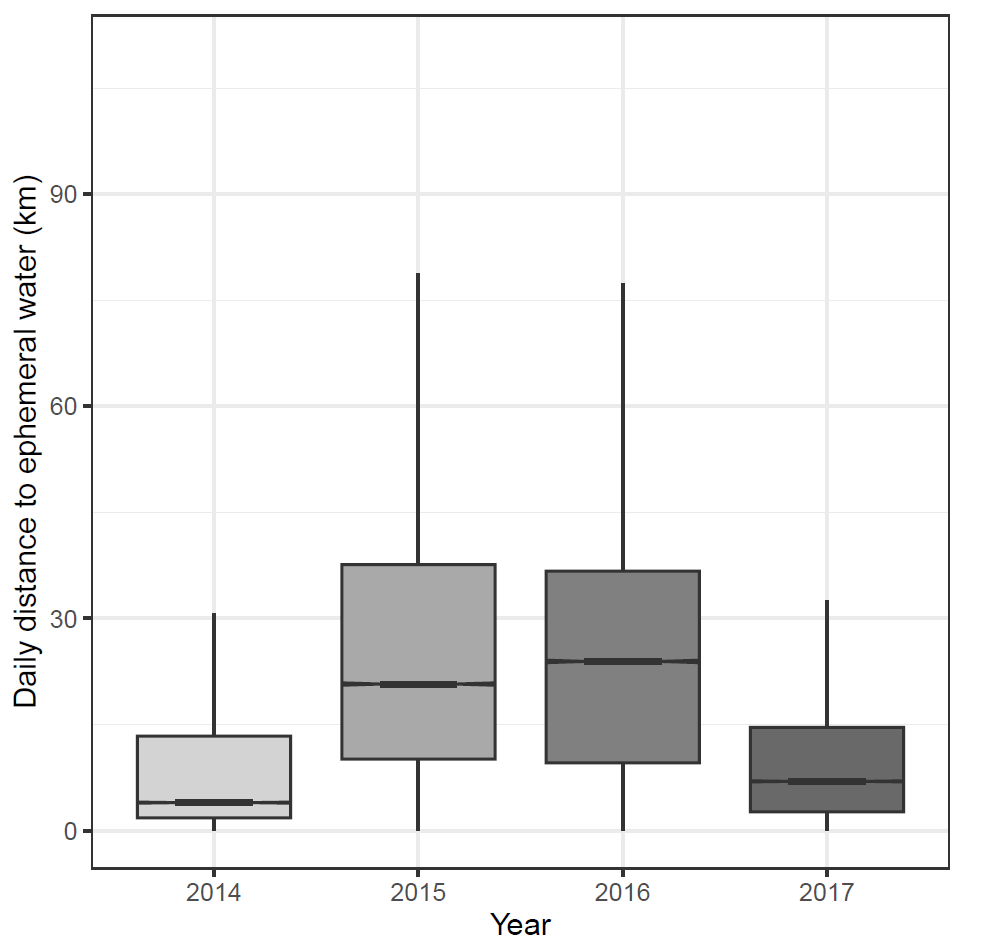


**Figure S6**


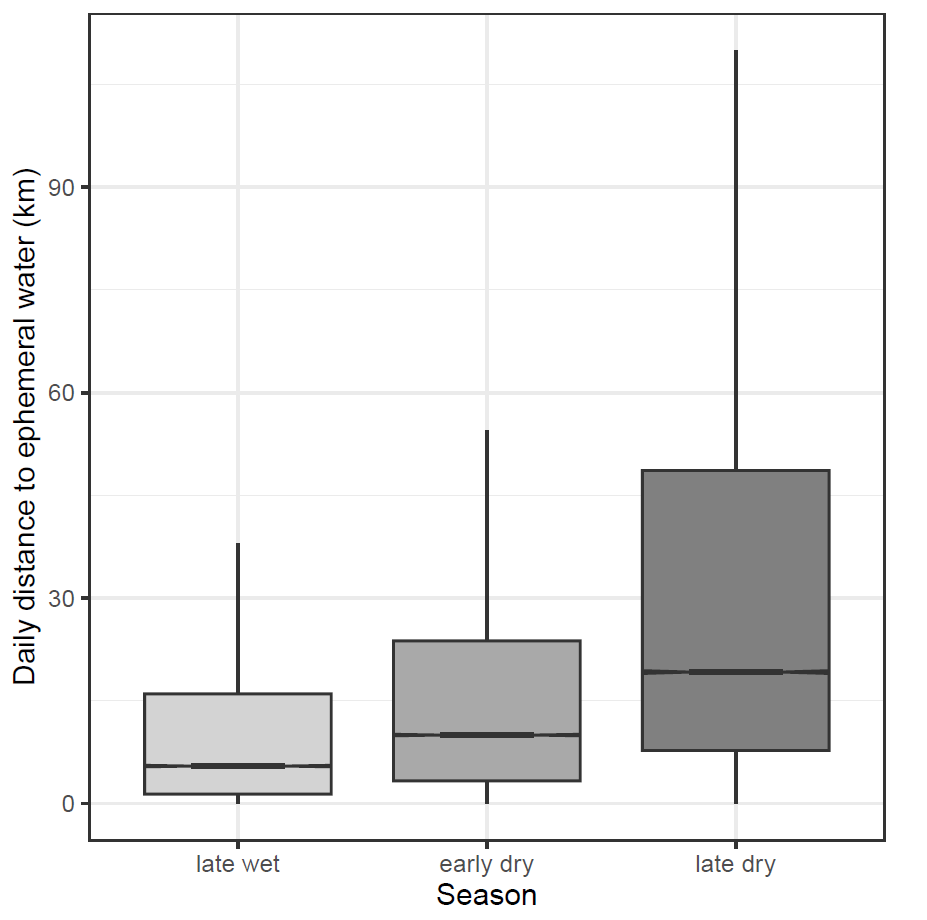


**Figure S7**


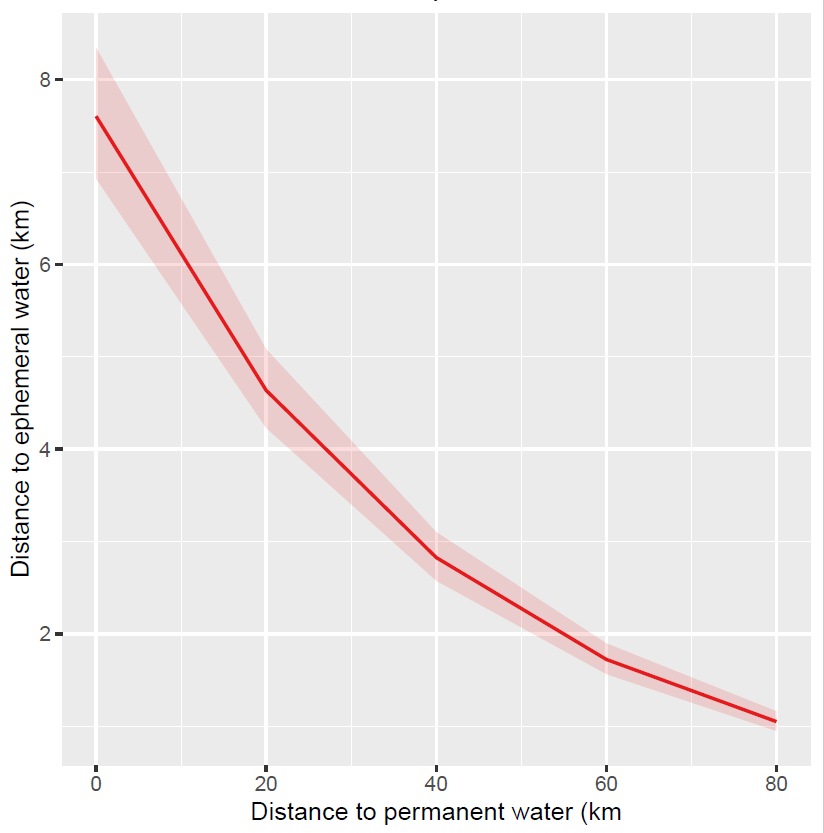


**Figure S8**

A

**Study area**


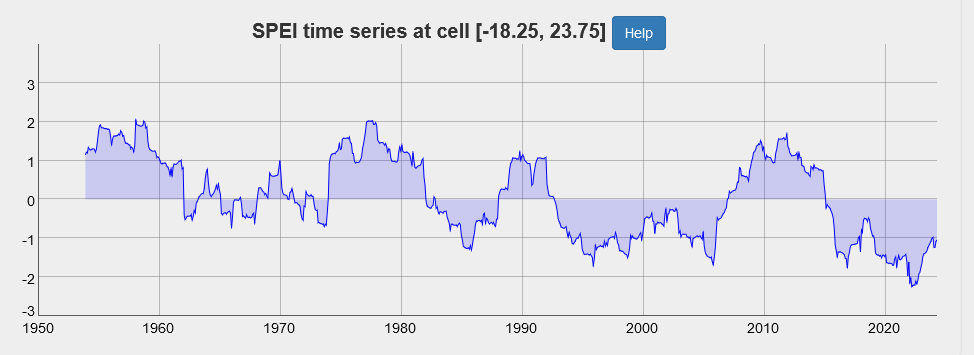


**SPEI**

**Year**

x

**Figure S9. A-B**
